# High-density Lipoprotein Cholesterol Is Negatively Correlated with Bone Mineral Density and Has Potential Predictive Value for Bone Loss

**DOI:** 10.1186/s12944-021-01497-7

**Published:** 2021-07-25

**Authors:** Yuchen Tang, Shenghong Wang, Qiong Yi, Yayi Xia, Bin Geng

**Affiliations:** 1 Department of Orthopaedics, Lanzhou University Second Hospital, #82 Cuiyingmen, Lanzhou, Gansu People’s Republic of China 730000; 2 Orthopaedics Key Laboratory of Gansu Province, Lanzhou, Gansu China; 3 Orthopaedic Clinical Research Center of Gansu Province, Lanzhou, Gansu China

**Keywords:** Bone mineral density, High-density lipoprotein cholesterol, Osteoporosis, Osteopenia

## Abstract

**Background:**

Many studies have shown that lipids play important roles in bone metabolism. However, the association between high-density lipoprotein cholesterol (HDL-C) and bone mineral density (BMD) is unclear. Therefore, this study aimed to investigate the linear or nonlinear relation between HDL-C levels and BMD and addressed whether the HDL-C levels had the potential values for predicting the risk of osteoporosis or osteopenia.

**Methods:**

Two researchers independently extracted all information from the National Health and Nutrition Examination Survey (NHANES) database. Participants over 20 years of age with available HDL-C and BMD data were enrolled in the final analysis. The linear relationship between HDL-C levels and BMD was assessed using multivariate linear regression models. Moreover, the nonlinear relationship was also characterized by fitted smoothing curves and generalized additive models. In addition, the odds ratio (OR) for osteopenia and osteoporosis was evaluated with multiple logistic regression models.

**Results:**

The weighted multivariable linear regression models demonstrated that HDL-C levels displayed an inverse association with BMD, especially among females and subjects aged 30 to 39 or 50 to 59. Moreover, the nonlinear relationship characterized by smooth curve fittings and generalized additive models suggested that (i) HDL-C levels displayed an inverted U-shaped relationship with BMD among women 30 to 39 or over 60 years of age; (ii) HDL-C levels exhibited a U-shaped association with BMD among women 20 to 29 or 50 to 59 years of age. In addition, females with high HDL levels (62-139 mg/dL) had an increased risk of osteopenia or osteoporosis.

**Conclusion:**

This study demonstrated that HDL-C levels exhibit an inverse correlation with BMD. Especially in females, clinicians need to be alert to patients with high HDL-C levels, which may indicate an increased risk of osteoporosis or osteopenia. For these patients, close monitoring of BMD and early intervention may be necessary.

**Supplementary Information:**

The online version contains supplementary material available at 10.1186/s12944-021-01497-7.

## Background

Lipids play critical roles in physiopathology and include a variety of substances. High-density lipoprotein cholesterol (HDL-C) is a ubiquitous molecule, and one type of cholesterol is contained in or bound to high-density lipoprotein (HDL) [1]. HDL-C is believed to have beneficial impacts on human health, and high HDL-C levels are considered to be better for preventing cardiovascular disease over a long time [2, 3]. For instance, Gordon et al. reported an independent inverse association between HDL-C levels and the rate of coronary artery disease [4]. Rosenson et al. observed that statin treatment, which can increase HDL-C levels, was beneficial in cardiovascular disease reduction [5]. However, over the past few years, different opinions have been presented. Madsen et al. reported that adults with extremely high HDL cholesterol levels (men: ≥ 116 mg/dL; women: ≥ 135 mg/dL) paradoxically have high all-cause mortality [6]. Hamer et al. observed that HDL-C levels and mortality presented a U-shaped relationship in participants in a large sample, demonstrating that subjects with high levels of HDL-C also had increased mortality [7]. These findings may indicate that researchers should reconsider the perspective on HDL-C.

Osteoporosis is a high-incidence chronic disease described as reduced bone mineral density (BMD) [8]. According to the International Osteoporosis Foundation, one-third of women and one-fifth of men over 50 years of age have osteoporosis or osteopenia and are at risk for osteoporotic fracture [9]. Simultaneously, as the population ages and grows, the prevalence of osteoporosis continues to rise [10]. At present, apart from genetic factors, age, or sex, the impact of other factors, such as lipid metabolism or lifestyle, on bone metabolism has recently attracted considerable concern [11–13]. Meanwhile, researchers hope to discover novel modalities for osteoporosis prevention and treatment.

At present, numerous studies have shown that lipids play important roles in bone metabolism [14–16]. For example, Li et al. demonstrated that statin drug treatment can increase BMD by lowering low-density lipoprotein cholesterol (LDL-C) levels [15]. In addition, Zheng et al. found that statins can increase total body BMD, and this effect was partly associated with lowering of LDL-C [16]. Moreover, it is uncertain and controversial whether HDL-C levels are correlated with BMD. There is some evidence that HDL-C levels are elevated in postmenopausal women and negatively associated with BMD. Maghbooli et al. found that HDL-C exhibited an inverse correlation with BMD in postmenopausal Iranian women with vitamin D deficiency [17]. Zhang et al. observed that HDL-C displayed a negative correlation with lumbar spine BMD in Chinese women [18]. Conversely, Cui et al. suggested that there was no association between HDL-C levels and BMD values at any sites in pre- and postmenopausal subjects [19]. Apart from the above, Jeong et al. observed that HDL-C exhibited a positive association with BMD in Korean postmenopausal women [20]. The conclusions of these studies remain controversial. Therefore, it is worth exploring the relation of HDL-C levels and BMD and determining whether HDL-C levels have potential value for predicting the risk of osteoporosis or osteopenia, which may provide a novel theoretical basis for understanding the aetiology of osteoporosis and developing treatments.

Accordingly, this study enrolled adults over 20 years of age and collected related information from the National Health and Nutrition Examination Survey (NHANES) database to explore the linear or nonlinear relationship between HDL-C and BMD and to investigate whether HDL-C levels have potential value for predicting the risk of osteoporosis or osteopenia.

## Materials and methods

### Study design and population

The NHANES database was compiled by the Centers for Disease Control, United States. The NHANES database collects and stores information on the health and nutritional status of American residents and is updated each year. The present study was a cross-sectional study. Two researchers (YT and SW) independently extracted data from NHANES 2005-2010 [21–23], and a third researcher (BG) regularly cross-checked the data collected. The ethics review board of the National Center for Health Statistics approved the study, and each participant signed a written informed consent form. Detailed information on the ethics application and written informed consent are provided on the NHANES website [24–26].

### Data collection

Two researchers (YT and SW) independently extracted the following information:

1. Demographic data [age, gender, race/ethnicity, education level, and income to poverty ratio]
2. Examination data [BMD of femoral regions (total femur; femur neck; trochanter; intertrochanter) and the lumbar (L) spine (total spine; L1; L2; L3; L4)]
3. Laboratory data [HDL-C level (mg/dL), alanine aminotransferase (ALT) (U/L), aspartate aminotransferase (AST) (U/L), cholesterol level (mg/dL), total calcium (mg/dL), and C-reactive protein (mg/dL)]
4. Questionnaire data [alcohol consumption status (had at least 12 alcohol drinks in the past one year), smoking status (smoked at least 100 cigarettes in life), BMI (derived from height and weight); diabetes (has a doctor told you that you have diabetes), and hypertension (ever been told you have high blood pressure)]
5. Weight value [According to the rules of the weight value selection provided on the NHANES website [27], “Full Sample Two-Year Mobile Examination Center Exam Weight (WTMEC2YR)” was selected to represent the weight value. The final weight value used for analysis was equal to one-third of the “Full Sample Two-Year Mobile Examination Center Exam Weight” due to combining three two-year cycles of the continuous NHANES]

### Inclusion and exclusion criteria

The inclusion criteria were: (1) participants over or equal to 20 years of age, and (2) participants with available BMD and HDL-C data. The exclusion criteria were as follows: (1) subjects with cancer or malignancy (have doctors told you had cancer or a malignancy?); (2) subjects who used female hormones (ever used female hormones, such as oestrogen or progesterone?); and (3) subjects missing other variables data (data missing, answered "do not know" and refused to answer were considered missing data) were excluded.

### Measurement of HDL-C levels

Briefly, based on the information provided on the NHANES website, the HDL-C measurement was performed at Lipid Laboratory, Johns Hopkins. Serum was collected for detection of HDL-C. Apolipoprotein-B (apoB)-containing lipoproteins were removed by reaction with blocking reagents and rendering them nonreactive with enzymatic cholesterol reagents under the assay conditions. HDL-C levels were measured using polyethylene glycol-coupled cholesteryl esterase, cholesterol oxidase, and sulfated alpha-cyclodextrin in the presence of Mg^2+^. Detailed information about the measurement of HDL-C is accessible on the NHANES website [28].

### Evaluation of BMD

BMD was evaluated using dual energy X-ray absorptiometry (DXA) scans. The sites of assessment included femoral regions (total femur; femur neck; trochanter; intertrochanter) and the lumbar spine (total spine; L1; L2; L3; L4). Health technologists who were certified radiology technologists conducted the DXA scans using a Hologic QDR 4500A instrument (Hologic, Inc., Bedford, MA, USA) and Apex software version 3.2. Further details of the DXA examination protocol are described in the Body Composition Procedures Manual provided on the NHANES website [29].

### Osteopenia and osteoporosis

According to a study by Looker et al. [30], the BMD reference value was the mean femoral BMD of non-Hispanic white men and women aged 20 to 29 years from the NHANES III database. Osteopenia was defined as a BMD value in any femoral region between -1 and -2.5 SD of the reference value, and osteoporosis was defined as a BMD value in any femoral region lower than -2.5 SD of the reference value. The specific values are presented in Supplementary Table S1.

### Statistical analysis

The baseline characteristics of all participants involved in the final analysis are described by the mean (continuity variable) or proportion (categorical variable). The linear relationship between HDL-C and BMD was assessed through weighted multivariate linear regression models. Subgroup analysis using multivariate linear regression models was performed to evaluate the linear relationship between HDL-C and BMD in diverse populations by stratifying age and sex. Moreover, the nonlinear relationship between HDL-C and BMD was characterized by smooth curve fittings and generalized additive models. The inflection point (if it existed) was calculated by employing two-piecewise linear regression models using a recursive algorithm. In addition, the odds ratio (OR) for osteopenia and osteoporosis was evaluated via multiple logistic regression analyses. *P* values less than 0.05 were defined as significant. All analyses were performed using R software, v.4.0.3 (Vienna, Austria: R Foundation for Statistical Computing, 2016) and EmpowerStats (version: 2.0. X&Y Solutions, Inc, Boston, MA. http://www.empowerstats. com). The frequency distribution graph of HDL-C was generated using Origin (version: 2021b. https://www.originlab.com/).

## Results

### Participant selection and baseline characteristics

**Fig. 1:**
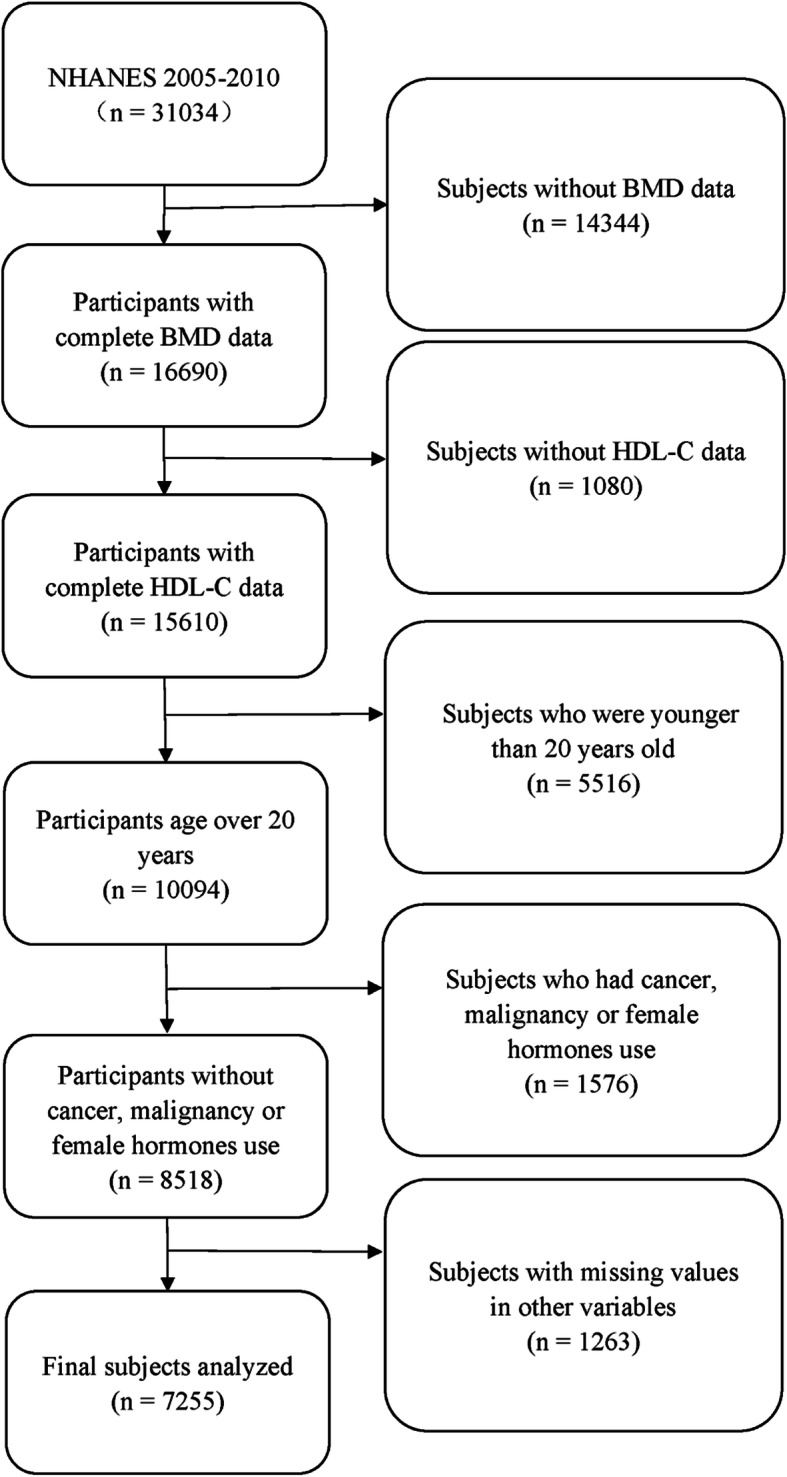
Flow chart of participants selection. NHANES, National Health and Nutrition Examination Survey; HDL-C, high-density lipoprotein cholesterol; BMD, bone mineral density

The information of 31,034 participants was extracted from the NHANES database 2005-2010. (i) Subjects without BMD data were excluded (*n* = 14344); (ii) subjects without HDL-C data were excluded (*n* = 1080); (iii) subjects below 20 years of age were excluded (*n* = 5516); (iv) subjects with cancer, malignancy or female hormone use were excluded (*n* = 1576); and (v) subjects with missing values for other variables were excluded (*n* = 1263, education level: 8, income to poverty ratio: 600, BMI: 244, smoking status: 1, drinking status: 349, hypertension: 8, diabetes: 4, ALT: 45, AST: 1, cholesterol: 1, C-reactive protein: 2). After that, 7,255 participants were enrolled in the final analysis. A flow chart of participant selection is shown in Fig. 1.

**Fig. 2:**
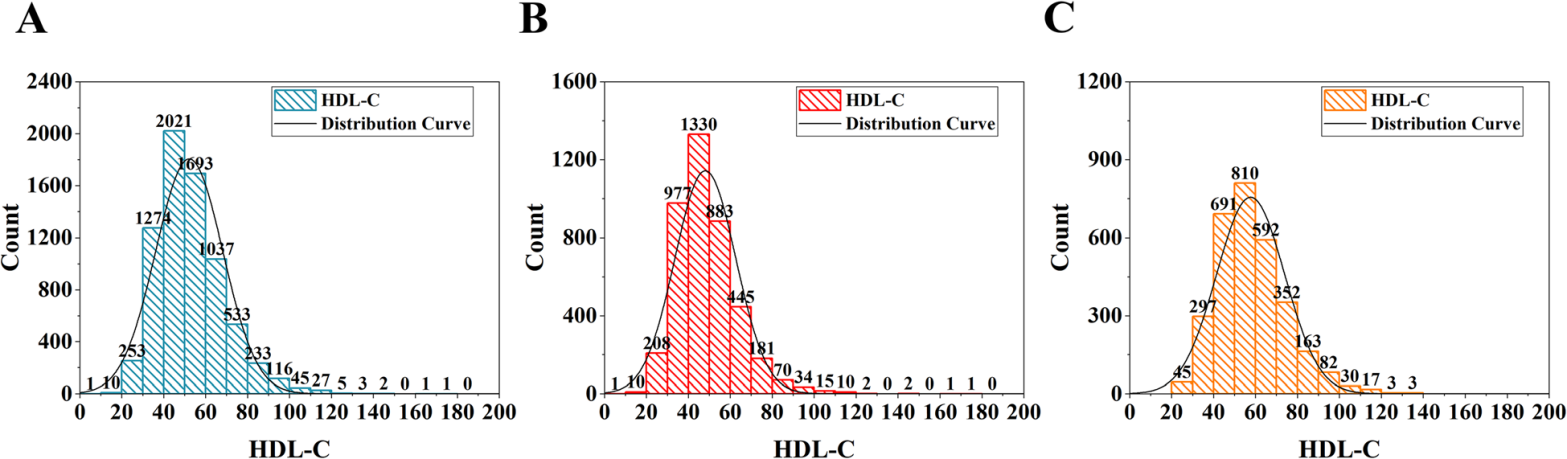
Distribution histogram of HDL-C. **a**. Among all participants; **b**. Among all males; **c**. Among all females. HDL-C, high-density lipoprotein cholesterol

Overall, the participants’ mean age was 41.74 ± 14.25 years, and most were males (56.93%) and non-Hispanic whites (69.43%). The majority of individuals had an above high school education level (59.85%) and a mean income to poverty ratio of 3.10 ± 1.63. Obesity (BMI ≥ 30), smoking (smoking at least 100 cigarettes in life), drinking (consuming at least 12 alcohol drinks past one year), hypertension, and diabetes accounted for 25.83%, 46.31%, 79.20%, 22.18%, and 5.38%, respectively. In addition, the mean ALT, AST, cholesterol, total calcium, and C-reactive protein levels were 26.48 ± 18.83, 25.75 ± 14.10, 9.46 ± 0.35, 196.71 ± 40.39, and 0.33 ± 0.68, respectively. The mean HDL-C level among all participants was 52.47 ± 15.94 mg/dL. In addition, the distribution of HDL-C, including among all participants, all males or all females, is presented in Fig. 2. The detailed results and other baseline characteristics are presented in Table 1.

**Table 1:** Weighted characteristics of the study population

Characteristics	Means or proportions
Age (years, mean ± SD)	41.74 ± 14.25
Sex, n (%)	
Male	4170 (56.93)
Female	3085 (43.07)
Race/ethnicity, n (%)	
Mexican American	1429 (8.85)
Other Hispanic	626 (4.71)
Non-Hispanic White	3397 (69.43)
Non-Hispanic Black	1445 (10.70)
Other Race	358 (6.30)
Education level, n (%)	
Under high school	1913 (17.14)
High school or equivalent	1662 (23.01)
Above high school	3680 (59.85)
Income to poverty ratio (mean ± SD)	3.10 ± 1.63
BMI, n (%)	
>=30	2008 (25.83)
>=25, <30	2704 (36.50)
<25	2543 (37.68)
Smoked at least 100 cigarettes in life, n (%)	
Yes	3394 (46.31)
No	3861 (53.69)
Had at least 12 alcohol drinks past one year? n (%)	
Yes	5462 (79.20)
No	1793 (20.80)
Hypertension, n (%)	
Yes	1844 (22.18)
No	5411 (77.82)
Diabetes, n (%)	
Yes	564 (5.38)
No	6590 (93.42)
Borderline	101 (1.21)
ALT (U/L, mean ± SD)	26.48 ± 18.83
AST (U/L, mean ± SD)	25.75 ± 14.10
Total calcium (mg/dL, mean ± SD)	9.46 ± 0.35
Cholesterol (mg/dL, mean ± SD)	196.71 ± 40.39
C-reactive protein (mg/dL, mean ± SD)	0.33 ± 0.68
HDL-C (mg/dL, mean ± SD)	52.47 ± 15.94
Total femur BMD (g/cm2, mean ± SD)	1.00 ± 0.15
Femur neck BMD (g/cm2, mean ± SD)	0.86 ± 0.14
Trochanter BMD (g/cm2, mean ± SD)	0.75 ± 0.13
Intertrochanter BMD (g/cm2, mean ± SD)	1.17 ± 0.18
Total spine BMD (g/cm2, mean ± SD)	1.05 ± 0.14
L1 BMD (g/cm2, mean ± SD)	0.97 ± 0.14
L2 BMD (g/cm2, mean ± SD)	1.06 ± 0.15
L3 BMD (g/cm2, mean ± SD)	1.08 ± 0.15
L4 BMD (g/cm2, mean ± SD)	1.08 ± 0.14

*ALT* alanine aminotransferase; *AST* aspartate aminotransferase; *HDL-C* high-density lipoprotein cholesterol; *BMI* body mass index; *SD* standard deviation; n, numbers of subjects; %, weighted percentage

### Relationship between HDL-C and BMD

**Fig. 3:**
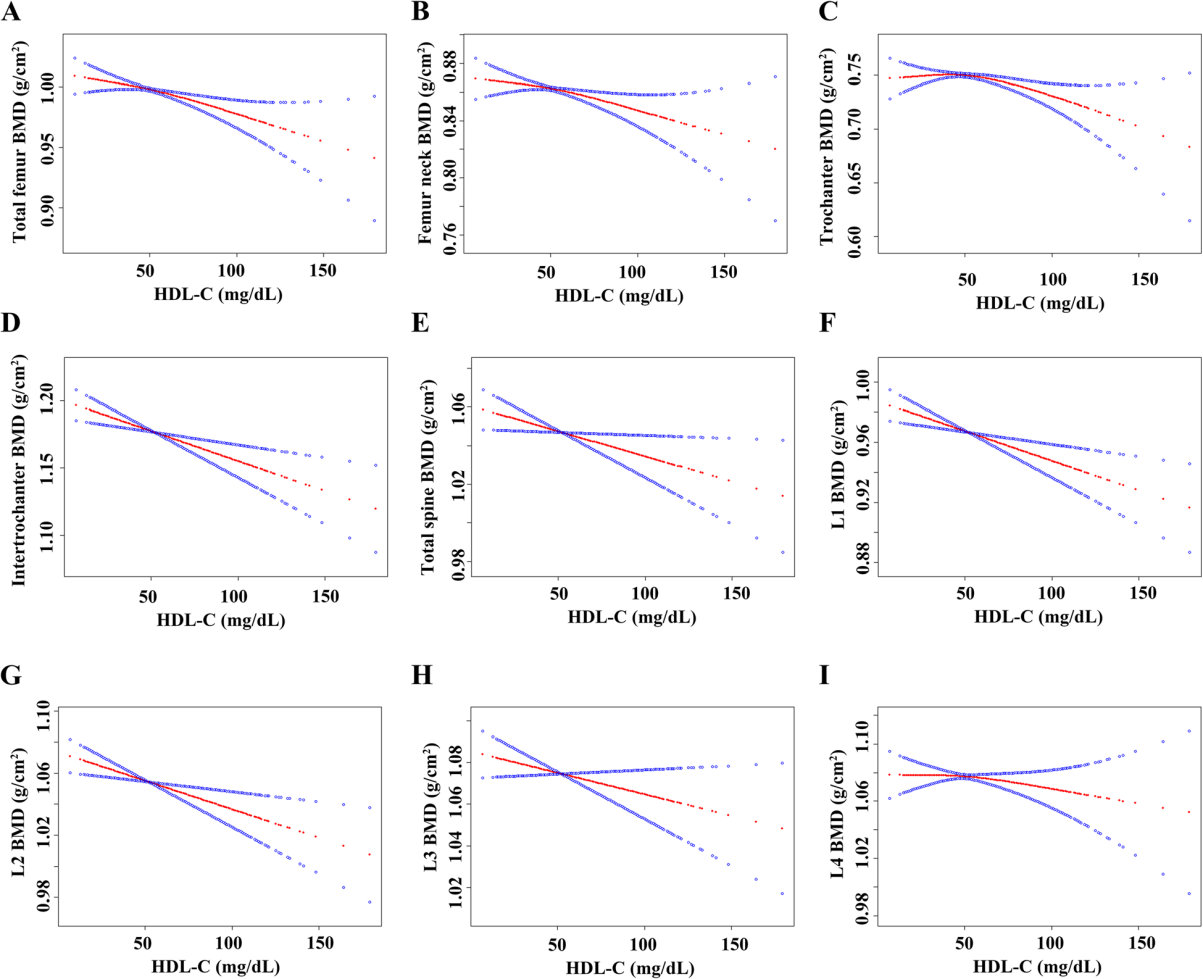
Association between HDL-C and BMD. Solid rad line represents the smooth curve fit between variables. Blue bands represent the 95% of confidence interval from the fit. Age, sex, race/ethnicity, education level, income to poverty ratio, BMI, smoking status, alcohol consumption status, hypertension, diabetes, ALT, AST, total calcium, cholesterol, and C-reactive protein were adjusted. **a**. Total femur BMD; **b**. Femur neck BMD; **c**. Trochanter BMD; **d**. Intertrochanter BMD; **e**. Total spine BMD; **f**. L1 BMD; **g**. L2 BMD; **h**. L3 BMD; **i**. L4 BMD. HDL-C, high-density lipoprotein cholesterol; BMD, bone mineral density; ALT, alanine aminotransferase; AST, aspartate aminotransferase

HDL-C levels displayed a negative association with BMD (*P* < 0.01) in Model 1 (no covariates were adjusted). Moreover, after adjusting for confounders (Model 2: age, sex, and race/ethnicity were adjusted; Model 3: age, sex, race/ethnicity, education level, income to poverty ratio, BMI, alcohol consumption status, smoking status, diabetes, hypertension, ALT, AST, total calcium, cholesterol, and C-reactive protein were adjusted), a negative association was still present and statistically significant. Moreover, a negative association was also observed in the nonlinear relationship between HDL-C levels and BMD assessed by smooth curve fittings and generalized additive models. The detailed results are displayed in Table 2 and Fig. 3.

**Table 2:** Association between HDL-C and BMD

	Model 1 β (95% CI) *P* value	Model 2 β (95% CI) *P* value	Model 3 β (95% CI) *P* value
Total femur BMD	-0.0023 (-0.0025, -0.0021) <0.000001	-0.0013 (-0.0015, -0.0011) <0.000001	-0.0004 (-0.0006, -0.0002) 0.000668
Femur neck BMD	-0.0017 (-0.0019, -0.0015) <0.000001	-0.0011 (-0.0013, -0.0009) <0.000001	-0.0003 (-0.0005, -0.0001) 0.004985
Trochanter BMD	-0.0016 (-0.0018, -0.0014) <0.000001	-0.0008 (-0.0010, -0.0007) <0.000001	-0.0002 (-0.0004, -0.0001) 0.012022
Intertrochanter BMD	-0.0027 (-0.0029, -0.0024) <0.000001	-0.0016 (-0.0018, -0.0013) <0.000001	-0.0005 (-0.0007, -0.0002) 0.000301
Total spine BMD	-0.0010 (-0.0012, -0.0008) <0.000001	-0.0010 (-0.0012, -0.0008) <0.000001	-0.0004 (-0.0006, -0.0002) 0.001002
L1 BMD	-0.0016 (-0.0018, -0.0014) <0.000001	-0.0012 (-0.0014, -0.0009) <0.000001	-0.0005 (-0.0007, -0.0002) 0.000112
L2 BMD	-0.0012 (-0.0014, -0.0010) <0.000001	-0.0011 (-0.0013, -0.0009) <0.000001	-0.0005 (-0.0007, -0.0003) 0.000030
L3 BMD	-0.0007 (-0.0009, -0.0005) <0.000001	-0.0009 (-0.0011, -0.0007) <0.000001	-0.0003 (-0.0006, -0.0001) 0.005108
L4 BMD	-0.0006 (-0.0008, -0.0004) <0.000001	-0.0008 (-0.0010, -0.0006) <0.000001	-0.0002 (-0.0005, -0.0000) 0.049655

Model 1: no covariates were adjusted. Model 2: age (20-29, 30-39, 40-49; 50-59; ≥60), sex (male; female), race/ethnicity (Mexican American; other Hispanic; non-Hispanic white; non-Hispanic black; other races) were adjusted. Model 3: age (20-29, 30-39, 40-49; 50-59; ≥60), sex (male; female), race/ethnicity (Mexican American; other Hispanic; non-Hispanic white; non-Hispanic black; other races), education level (under high school; high school or equivalent; above high school), income to poverty ratio (quartile groups), BMI (obese, overweight, normal), smoking status (less than 100 cigarettes; greater than or equal to 100 cigarettes), alcohol consumption status (had at least 12 alcohol drinks past one year; have less than 12 alcohol drinks past one year), hypertension (yes; no), diabetes (yes; no; borderline), ALT (quartile groups), AST (quartile groups), total calcium (quartile groups), cholesterol (quartile groups), and C-reactive protein (quartile groups) were adjusted. *HDL-C* high-density lipoprotein cholesterol; *BMD* bone mineral density; *ALT* alanine aminotransferase; *AST* aspartate aminotransferase

### Subgroup analysis

After adjusting for covariates, the results of subgroup analysis showed that the association between HDL-C levels and BMD was mainly present in females or participants aged 30 to 39 or 50-59 years. Detailed information on the subgroup analysis is shown in **Tables** **3****-****4**.

**Table 3:** Association between HDL-C and BMD stratified by age

		Model 1 β (95% CI) *P* value	Model 2 β (95% CI) *P* value	Model 3 β (95% CI) *P* value
Total femur BMD	20≤Aged<30	-0.0018 (-0.0023, -0.0013) <0.000001	-0.0008 (-0.0013, -0.0004) 0.000480	0.0001 (-0.0004, 0.0006) 0.598614
	30≤Aged<40	-0.0023 (-0.0027, -0.0018) <0.000001	-0.0016 (-0.0020, -0.0012) <0.000001	-0.0009 (-0.0013, -0.0004) 0.000185
	40≤Aged<50	-0.0019 (-0.0023, -0.0016) <0.000001	-0.0012 (-0.0015, -0.0008) <0.000001	-0.0002 (-0.0006, 0.0002) 0.281833
	50≤Aged<60	-0.0027 (-0.0032, -0.0021) <0.000001	-0.0018 (-0.0023, -0.0013) <0.000001	-0.0009 (-0.0014, -0.0003) 0.002075
	60≤Aged	-0.0029 (-0.0035, -0.0023) <0.000001	-0.0012 (-0.0017, -0.0007) 0.000012	-0.0003 (-0.0008, 0.0003) 0.327776
Femur neck BMD	20≤Aged<30	-0.0014 (-0.0018, -0.0009) <0.000001	-0.0007 (-0.0012, -0.0003) 0.001761	0.0002 (-0.0003, 0.0007) 0.335319
	30≤Aged<40	-0.0015 (-0.0019, -0.0011) <0.000001	-0.0013 (-0.0017, -0.0009) <0.000001	-0.0006 (-0.0011, -0.0002) 0.008064
	40≤Aged<50	-0.0013 (-0.0017, -0.0010) <0.000001	-0.0011 (-0.0014, -0.0007) <0.000001	-0.0003 (-0.0007, 0.0001) 0.149013
	50≤Aged<60	-0.0019 (-0.0023, -0.0015) <0.000001	-0.0015 (-0.0020, -0.0011) <0.000001	-0.0007 (-0.0012, -0.0001) 0.011229
	60≤Aged	-0.0020 (-0.0025, -0.0015) <0.000001	-0.0011 (-0.0016, -0.0006) 0.000009	-0.0003 (-0.0008, 0.0002) 0.195810
Trochanter BMD	20≤Aged<30	-0.0013 (-0.0018, -0.0009) <0.000001	-0.0005 (-0.0009, -0.0001) 0.008268	-0.0000 (-0.0005, 0.0004) 0.936942
	30≤Aged<40	-0.0015 (-0.0019, -0.0012) <0.000001	-0.0010 (-0.0014, -0.0006) <0.000001	-0.0006 (-0.0010, -0.0002) 0.007441
	40≤Aged<50	-0.0013 (-0.0017, -0.0010) <0.000001	-0.0007 (-0.0011, -0.0004) 0.000022	-0.0002 (-0.0005, 0.0002) 0.342789
	50≤Aged<60	-0.0020 (-0.0025, -0.0016) <0.000001	-0.0013 (-0.0018, -0.0008) <0.000001	-0.0007 (-0.0012, -0.0001) 0.013165
	60≤Aged	-0.0021 (-0.0026, -0.0016) <0.000001	-0.0006 (-0.0010, -0.0001) 0.011620	0.0000 (-0.0004, 0.0005) 0.930179
Intertrochanter BMD	20≤Aged<30	-0.0021 (-0.0027, -0.0016) <0.000001	-0.0010 (-0.0015, -0.0005) 0.000211	0.0001 (-0.0005, 0.0007) 0.697817
	30≤Aged<40	-0.0027 (-0.0032, -0.0022) <0.000001	-0.0019 (-0.0024, -0.0014) <0.000001	-0.0011 (-0.0016, -0.0006) 0.000067
	40≤Aged<50	-0.0023 (-0.0028, -0.0019) <0.000001	-0.0014 (-0.0018, -0.0009) <0.000001	-0.0002 (-0.0007, 0.0003) 0.365447
	50≤Aged<60	-0.0031 (-0.0037, -0.0025) <0.000001	-0.0021 (-0.0028, -0.0015) <0.000001	-0.0010 (-0.0017, -0.0004) 0.002512
	60≤Aged	-0.0035 (-0.0042, -0.0029) <0.000001	-0.0016 (-0.0022, -0.0009) 0.000002	-0.0005 (-0.0011, 0.0002) 0.160472
Total spine BMD	20≤Aged<30	-0.0005 (-0.0009, -0.0002) 0.006856	-0.0007 (-0.0011, -0.0003) 0.000465	-0.0001 (-0.0006, 0.0003) 0.540391
	30≤Aged<40	-0.0005 (-0.0009, -0.0001) 0.021297	-0.0009 (-0.0013, -0.0005) 0.000009	-0.0006 (-0.0010, -0.0001) 0.012320
	40≤Aged<50	-0.0004 (-0.0007, -0.0000) 0.037080	-0.0007 (-0.0011, -0.0003) 0.000666	-0.0001 (-0.0005, 0.0004) 0.795368
	50≤Aged<60	-0.0021 (-0.0026, -0.0016) <0.000001	-0.0019 (-0.0024, -0.0013) <0.000001	-0.0012 (-0.0018, -0.0005) 0.000354
	60≤Aged	-0.0024 (-0.0030, -0.0018) <0.000001	-0.0010 (-0.0016, -0.0004) 0.000661	-0.0004 (-0.0010, 0.0002) 0.171588
L1 BMD	20≤Aged<30	-0.0009 (-0.0013, -0.0004) 0.000068	-0.0006 (-0.0011, -0.0002) 0.003937	0.0001 (-0.0004, 0.0006) 0.734575
	30≤Aged<40	-0.0011 (-0.0015, -0.0007) <0.000001	-0.0010 (-0.0015, -0.0006) 0.000003	-0.0006 (-0.0011, -0.0001) 0.015966
	40≤Aged<50	-0.0011 (-0.0015, -0.0008) <0.000001	-0.0009 (-0.0013, -0.0005) 0.000013	-0.0002 (-0.0007, 0.0002) 0.357967
	50≤Aged<60	-0.0028 (-0.0033, -0.0022) <0.000001	-0.0022 (-0.0027, -0.0016) <0.000001	-0.0014 (-0.0020, -0.0008) 0.000018
	60≤Aged	-0.0033 (-0.0039, -0.0026) <0.000001	-0.0014 (-0.0020, -0.0009) <0.000001	-0.0007 (-0.0013, -0.0001) 0.020440
L2 BMD	20≤Aged<30	-0.0007 (-0.0011, -0.0003) 0.001453	-0.0008 (-0.0012, -0.0003) 0.000564	-0.0001 (-0.0006, 0.0003) 0.585766
	30≤Aged<40	-0.0007 (-0.0011, -0.0003) 0.001105	-0.0011 (-0.0015, -0.0006) 0.000003	-0.0007 (-0.0012, -0.0002) 0.005244
	40≤Aged<50	-0.0006 (-0.0010, -0.0002) 0.001416	-0.0008 (-0.0012, -0.0004) 0.000099	-0.0002 (-0.0007, 0.0002) 0.353490
	50≤Aged<60	-0.0023 (-0.0029, -0.0017) <0.000001	-0.0020 (-0.0026, -0.0014) <0.000001	-0.0014 (-0.0021, -0.0007) 0.000065
	60≤Aged	-0.0027 (-0.0033, -0.0021) <0.000001	-0.0011 (-0.0017, -0.0005) 0.000327	-0.0006 (-0.0012, 0.0001) 0.079934
L3 BMD	20≤Aged<30	-0.0003 (-0.0008, 0.0001) 0.106832	-0.0007 (-0.0012, -0.0003) 0.000815	-0.0002 (-0.0007, 0.0002) 0.312215
	30≤Aged<40	-0.0001 (-0.0005, 0.0003) 0.650876	-0.0009 (-0.0013, -0.0004) 0.000094	-0.0006 (-0.0011, -0.0001) 0.012140
	40≤Aged<50	0.0001 (-0.0003, 0.0005) 0.712982	-0.0005 (-0.0009, -0.0001) 0.027419	0.0001 (-0.0004, 0.0005) 0.761201
	50≤Aged<60	-0.0019 (-0.0025, -0.0013) <0.000001	-0.0019 (-0.0025, -0.0013) <0.000001	-0.0013 (-0.0020, -0.0006) 0.000341
	60≤Aged	-0.0022 (-0.0028, -0.0015) <0.000001	-0.0009 (-0.0015, -0.0002) 0.007370	-0.0002 (-0.0009, 0.0005) 0.588529
L4 BMD	20≤Aged<30	-0.0004 (-0.0008, 0.0000) 0.060890	-0.0008 (-0.0012, -0.0003) 0.000663	-0.0002 (-0.0007, 0.0003) 0.373641
	30≤Aged<40	-0.0001 (-0.0005, 0.0003) 0.679656	-0.0008 (-0.0012, -0.0003) 0.000554	-0.0004 (-0.0009, 0.0001) 0.086672
	40≤Aged<50	-0.0001 (-0.0005, 0.0003) 0.712879	-0.0006 (-0.0010, -0.0002) 0.006600	0.0001 (-0.0004, 0.0005) 0.810854
	50≤Aged<60	-0.0017 (-0.0022, -0.0011) <0.000001	-0.0016 (-0.0022, -0.0010) <0.000001	-0.0008 (-0.0014, -0.0001) 0.029822
	60≤Aged	-0.0019 (-0.0026, -0.0013) <0.000001	-0.0007 (-0.0014, -0.0001) 0.022931	-0.0003 (-0.0010, 0.0004) 0.399114

Model 1: no covariates were adjusted. Model 2: sex (male; female) and race/ethnicity (Mexican American; other Hispanic; non-Hispanic white; non-Hispanic black; other Races) were adjusted. Model 3: sex (male; female), race/ethnicity (Mexican American; other Hispanic; non-Hispanic white; non-Hispanic black; other races), education level (under high school; high school or equivalent; above high school), income to poverty ratio (quartile groups), BMI (obese, overweight, normal), smoking status (less than 100 cigarettes; greater than or equal to 100 cigarettes), alcohol consumption status (had at least 12 alcohol drinks past one year; have less than 12 alcohol drinks past one year), hypertension (yes; no), diabetes (yes; no; borderline), ALT (quartile groups), AST (quartile groups), total calcium (quartile groups), cholesterol (quartile groups), and C-reactive protein (quartile groups) were adjusted. *HDL-C* high-density lipoprotein cholesterol; *BMD* bone mineral density; *ALT* alanine aminotransferase; *AST* aspartate aminotransferase

**Table 4:** Association between HDL-C and BMD stratified by sex

		Model 1 β (95% CI) *P* value	Model 2 β (95% CI) *P* value	Model 3 β (95% CI) *P* value
Total femur BMD	Male	-0.0011 (-0.0014, -0.0008) <0.000001	-0.0012 (-0.0015, -0.0009) <0.000001	-0.0002 (-0.0006, 0.0001) 0.116880
	Female	-0.0015 (-0.0018, -0.0012) <0.000001	-0.0014 (-0.0017, -0.0012) <0.000001	-0.0005 (-0.0008, -0.0002) 0.000302
Femur neck BMD	Male	-0.0009 (-0.0012, -0.0006) <0.000001	-0.0010 (-0.0012, -0.0007) <0.000001	-0.0001 (-0.0004, 0.0002) 0.449640
	Female	-0.0015 (-0.0018, -0.0012) <0.000001	-0.0013 (-0.0016, -0.0011) <0.000001	-0.0005 (-0.0008, -0.0002) 0.000374
Trochanter BMD	Male	-0.0006 (-0.0008, -0.0003) 0.000047	-0.0007 (-0.0010, -0.0004) <0.000001	-0.0001 (-0.0004, 0.0002) 0.422344
	Female	-0.0010 (-0.0013, -0.0008) <0.000001	-0.0010 (-0.0012, -0.0008) <0.000001	-0.0004 (-0.0007, -0.0002) 0.001635
Intertrochanter BMD	Male	-0.0013 (-0.0017, -0.0010) <0.000001	-0.0015 (-0.0018, -0.0011) <0.000001	-0.0003 (-0.0007, 0.0000) 0.063777
	Female	-0.0018 (-0.0021, -0.0015) <0.000001	-0.0017 (-0.0020, -0.0014) <0.000001	-0.0006 (-0.0010, -0.0003) 0.000359
Total spine BMD	Male	-0.0005 (-0.0008, -0.0002) 0.002365	-0.0007 (-0.0010, -0.0004) 0.000003	-0.0001 (-0.0004, 0.0002) 0.520811
	Female	-0.0013 (-0.0016, -0.0010) <0.000001	-0.0013 (-0.0015, -0.0010) <0.000001	-0.0007 (-0.0010, -0.0004) 0.000009
L1 BMD	Male	-0.0006 (-0.0009, -0.0003) 0.000064	-0.0008 (-0.0011, -0.0005) <0.000001	-0.0001 (-0.0004, 0.0003) 0.676951
	Female	-0.0016 (-0.0019, -0.0012) <0.000001	-0.0015 (-0.0018, -0.0012) <0.000001	-0.0008 (-0.0012, -0.0005) <0.000001
L2 BMD	Male	-0.0006 (-0.0009, -0.0003) 0.000265	-0.0008 (-0.0011, -0.0005) <0.000001	-0.0003 (-0.0006, 0.0001) 0.135526
	Female	-0.0014 (-0.0017, -0.0011) <0.000001	-0.0014 (-0.0017, -0.0011) <0.000001	-0.0008 (-0.0011, -0.0005) 0.000003
L3 BMD	Male	-0.0004 (-0.0007, -0.0000) 0.027488	-0.0006 (-0.0009, -0.0003) 0.000127	-0.0001 (-0.0004, 0.0002) 0.584406
	Female	-0.0012 (-0.0015, -0.0009) <0.000001	-0.0012 (-0.0015, -0.0009) <0.000001	-0.0007 (-0.0010, -0.0003) 0.000073
L4 BMD	Male	-0.0003 (-0.0006, 0.0000) 0.059045	-0.0006 (-0.0009, -0.0003) 0.000131	0.0000 (-0.0003, 0.0004) 0.962035
	Female	-0.0011 (-0.0014, -0.0007) <0.000001	-0.0011 (-0.0014, -0.0008) <0.000001	-0.0006 (-0.0009, -0.0002) 0.000690

Model 1: no covariates were adjusted. Model 2: age (20-29, 30-39, 40-49; 50-59; ≥60) and race/ethnicity (Mexican American; other Hispanic; non-Hispanic white; non-Hispanic black; other races) were adjusted. Model 3: age (20-29, 30-39, 40-49; 50-59; ≥60), race/ethnicity (Mexican American; other Hispanic; non-Hispanic white; non-Hispanic black; other races), education level (under high school; high school or equivalent; above high school), income to poverty ratio (quartile groups), BMI (obese, overweight, normal), smoking status (less than 100 cigarettes; greater than or equal to 100 cigarettes), alcohol consumption status (had at least 12 alcohol drinks past one year; have less than 12 alcohol drinks past one year), hypertension (yes; no), diabetes (yes; no; borderline), ALT (quartile groups), AST (quartile groups), total calcium (quartile groups), cholesterol (quartile groups), and C-reactive protein (quartile groups) were adjusted. *HDL-C* high-density lipoprotein cholesterol; *BMD* bone mineral density; *ALT* alanine aminotransferase; *AST* aspartate aminotransferase

**Fig. 4:**
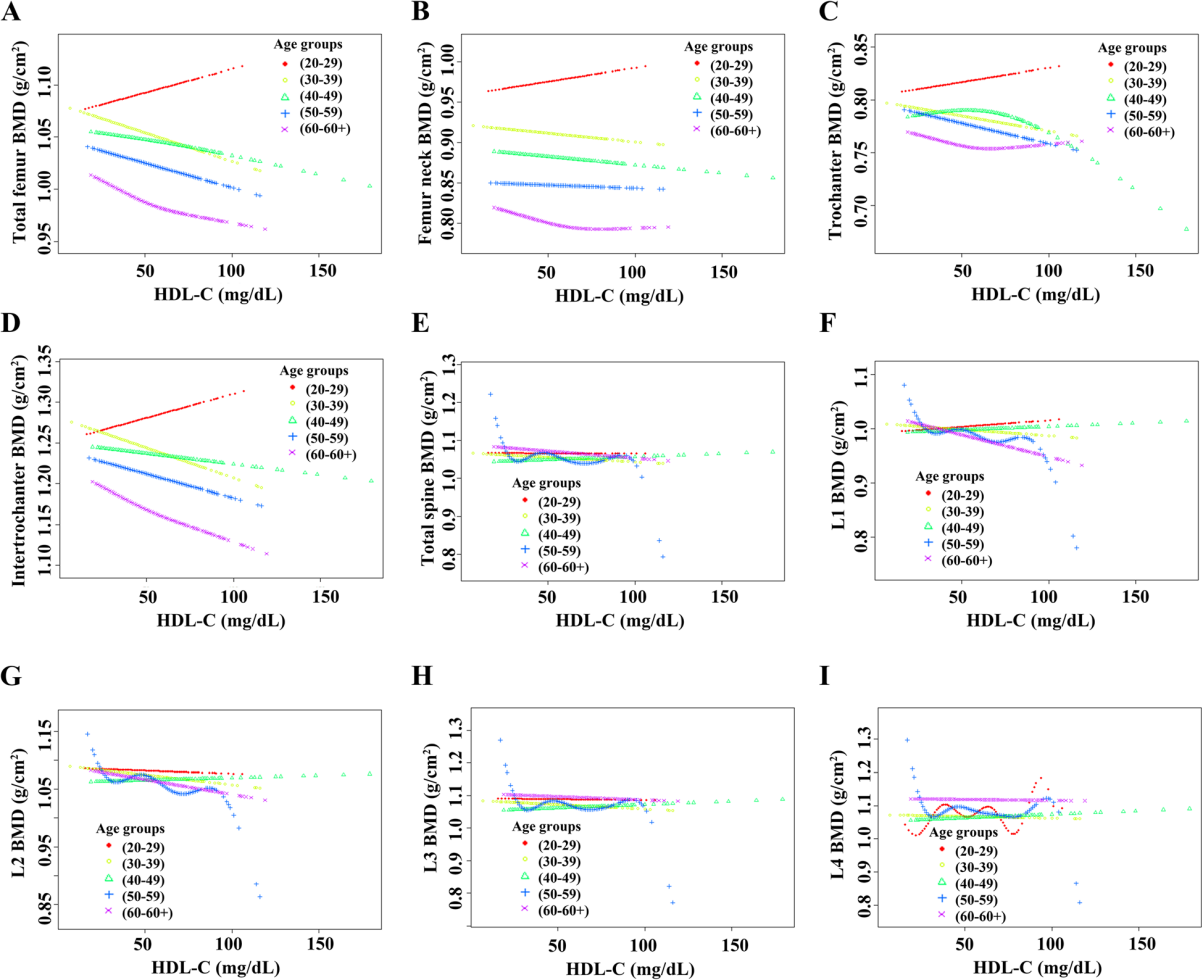
Association between HDL-C and BMD in male participants. Race/ethnicity, education level, income to poverty ratio, BMI, smoking status, alcohol consumption status, hypertension, diabetes, ALT, AST, total calcium, cholesterol, and C-reactive protein were adjusted. **a**. Total femur BMD; **b**. Femur neck BMD; **c**. Trochanter BMD; **d**. Intertrochanter BMD; **e**. Total spine BMD; **f**. L1 BMD; **g**. L2 BMD; **h**. L3 BMD; **i**. L4 BMD. Red line: 20≤Aged<30; Yellow line: 30≤Aged<40; Green line: 40≤Aged<50; Blue line: 50≤Aged<60; Purple line: 60≤Aged. HDL-C, high-density lipoprotein cholesterol; BMD, bone mineral density; ALT, alanine aminotransferase; AST, aspartate aminotransferase

For males, HDL-C levels exhibited an inverse association with BMD in Model 1 and Model 2. However, when all covariates were adjusted, this relationship was not present. In addition, when the nonlinear relationship was characterized by smooth curve fittings and generalized additive models, the inverse correlation between HDL-C levels and BMD did not survive in most groups. The detailed results are listed in Table 5 and Fig. 4.

**Table 5:** Association between HDL-C and BMD in males

		Model 1 β (95% CI) *P* value	Model 2 β (95% CI) *P* value	Model 3 β (95% CI) *P* value
Total femur BMD	20≤Aged<30	-0.0006 (-0.0013, 0.0001) 0.118476	-0.0009 (-0.0016, -0.0001) 0.017990	0.0001 (-0.0007, 0.0009) 0.776354
	30≤Aged<40	-0.0014 (-0.0021, -0.0008) 0.000015	-0.0015 (-0.0022, -0.0009) 0.000003	-0.0008 (-0.0015, -0.0001) 0.019154
	40≤Aged<50	-0.0007 (-0.0012, -0.0001) 0.028729	-0.0009 (-0.0015, -0.0003) 0.003117	0.0001 (-0.0006, 0.0007) 0.820437
	50≤Aged<60	-0.0013 (-0.0021, -0.0006) 0.000698	-0.0017 (-0.0024, -0.0009) 0.000026	-0.0005 (-0.0014, 0.0003) 0.203276
	60≤Aged	-0.0011 (-0.0018, -0.0004) 0.002495	-0.0012 (-0.0019, -0.0005) 0.000605	-0.0004 (-0.0011, 0.0003) 0.220103
Femur neck BMD	20≤Aged<30	-0.0005 (-0.0012, 0.0002) 0.165831	-0.0008 (-0.0015, -0.0001) 0.032699	0.0002 (-0.0006, 0.0010) 0.639104
	30≤Aged<40	-0.0010 (-0.0017, -0.0004) 0.002135	-0.0011 (-0.0017, -0.0005) 0.000712	-0.0004 (-0.0011, 0.0002) 0.198245
	40≤Aged<50	-0.0006 (-0.0012, -0.0001) 0.032023	-0.0009 (-0.0014, -0.0003) 0.001909	-0.0001 (-0.0007, 0.0005) 0.673213
	50≤Aged<60	-0.0008 (-0.0014, -0.0001) 0.021113	-0.0011 (-0.0017, -0.0004) 0.001188	0.0000 (-0.0007, 0.0007) 0.906714
	60≤Aged	-0.0009 (-0.0015, -0.0002) 0.007393	-0.0011 (-0.0017, -0.0004) 0.001304	-0.0004 (-0.0010, 0.0003) 0.281805
Trochanter BMD	20≤Aged<30	-0.0003 (-0.0009, 0.0003) 0.348101	-0.0005 (-0.0011, 0.0001) 0.113008	-0.0001 (-0.0008, 0.0006) 0.827111
	30≤Aged<40	-0.0008 (-0.0014, -0.0003) 0.004504	-0.0009 (-0.0014, -0.0003) 0.001459	-0.0004 (-0.0010, 0.0002) 0.155924
	40≤Aged<50	-0.0003 (-0.0008, 0.0002) 0.278418	-0.0004 (-0.0010, 0.0001) 0.101470	0.0001 (-0.0005, 0.0007) 0.672573
	50≤Aged<60	-0.0009 (-0.0016, -0.0002) 0.009191	-0.0012 (-0.0019, -0.0005) 0.000699	-0.0004 (-0.0011, 0.0004) 0.364990
	60≤Aged	-0.0004 (-0.0010, 0.0002) 0.201940	-0.0005 (-0.0011, 0.0001) 0.106856	-0.0001 (-0.0007, 0.0005) 0.713744
Intertrochanter BMD	20≤Aged<30	-0.0007 (-0.0016, 0.0001) 0.094572	-0.0011 (-0.0019, -0.0002) 0.011121	0.0001 (-0.0008, 0.0010) 0.769779
	30≤Aged<40	-0.0018 (-0.0026, -0.0011) 0.000003	-0.0019 (-0.0027, -0.0012) <0.000001	-0.0011 (-0.0019, -0.0003) 0.005767
	40≤Aged<50	-0.0008 (-0.0015, -0.0001) 0.022436	-0.0011 (-0.0018, -0.0004) 0.002459	0.0001 (-0.0006, 0.0009) 0.769447
	50≤Aged<60	-0.0016 (-0.0025, -0.0007) 0.000748	-0.0019 (-0.0028, -0.0010) 0.000042	-0.0007 (-0.0016, 0.0003) 0.190718
	60≤Aged	-0.0015 (-0.0023, -0.0007) 0.000390	-0.0017 (-0.0025, -0.0008) 0.000101	-0.0007 (-0.0015, 0.0001) 0.102944
Total spine BMD	20≤Aged<30	-0.0002 (-0.0009, 0.0004) 0.432167	-0.0005 (-0.0011, 0.0002) 0.141290	0.0000 (-0.0007, 0.0007) 0.984658
	30≤Aged<40	-0.0006 (-0.0012, -0.0000) 0.045489	-0.0007 (-0.0013, -0.0001) 0.021858	-0.0002 (-0.0009, 0.0005) 0.545599
	40≤Aged<50	0.0000 (-0.0006, 0.0006) 0.894495	-0.0002 (-0.0008, 0.0004) 0.520570	0.0006 (-0.0001, 0.0012) 0.086857
	50≤Aged<60	-0.0013 (-0.0021, -0.0005) 0.001560	-0.0017 (-0.0025, -0.0009) 0.000021	-0.0007 (-0.0016, 0.0002) 0.109719
	60≤Aged	-0.0006 (-0.0013, 0.0002) 0.140196	-0.0007 (-0.0015, 0.0000) 0.051753	-0.0006 (-0.0014, 0.0002) 0.150883
L1 BMD	20≤Aged<30	-0.0002 (-0.0008, 0.0005) 0.627418	-0.0003 (-0.0010, 0.0003) 0.298880	0.0003 (-0.0004, 0.0010) 0.458371
	30≤Aged<40	-0.0005 (-0.0012, 0.0001) 0.086657	-0.0006 (-0.0012, 0.0000) 0.053243	-0.0001 (-0.0007, 0.0006) 0.858850
	40≤Aged<50	-0.0001 (-0.0008, 0.0005) 0.668590	-0.0003 (-0.0010, 0.0003) 0.274953	0.0005 (-0.0002, 0.0012) 0.125866
	50≤Aged<60	-0.0015 (-0.0023, -0.0007) 0.000174	-0.0019 (-0.0027, -0.0011) 0.000002	-0.0008 (-0.0016, 0.0001) 0.075641
	60≤Aged	-0.0011 (-0.0018, -0.0004) 0.002960	-0.0012 (-0.0019, -0.0005) 0.001050	-0.0008 (-0.0016, -0.0000) 0.037263
L2 BMD	20≤Aged<30	-0.0003 (-0.0010, 0.0003) 0.348527	-0.0005 (-0.0012, 0.0001) 0.117012	-0.0001 (-0.0008, 0.0007) 0.828919
	30≤Aged<40	-0.0007 (-0.0013, -0.0000) 0.046582	-0.0007 (-0.0014, -0.0001) 0.023423	-0.0002 (-0.0009, 0.0005) 0.535701
	40≤Aged<50	-0.0001 (-0.0007, 0.0005) 0.815957	-0.0003 (-0.0009, 0.0003) 0.338671	0.0004 (-0.0003, 0.0011) 0.221228
	50≤Aged<60	-0.0015 (-0.0023, -0.0007) 0.000477	-0.0019 (-0.0027, -0.0011) 0.000008	-0.0011 (-0.0020, -0.0001) 0.024255
	60≤Aged	-0.0005 (-0.0013, 0.0002) 0.180331	-0.0007 (-0.0015, 0.0001) 0.075794	-0.0006 (-0.0014, 0.0002) 0.156083
L3 BMD	20≤Aged<30	-0.0002 (-0.0009, 0.0004) 0.483580	-0.0005 (-0.0011, 0.0002) 0.173354	-0.0001 (-0.0009, 0.0006) 0.722289
	30≤Aged<40	-0.0006 (-0.0013, 0.0000) 0.061670	-0.0007 (-0.0014, -0.0001) 0.029734	-0.0003 (-0.0011, 0.0004) 0.346774
	40≤Aged<50	0.0002 (-0.0005, 0.0008) 0.566619	-0.0001 (-0.0007, 0.0006) 0.870392	0.0006 (-0.0001, 0.0013) 0.104112
	50≤Aged<60	-0.0012 (-0.0021, -0.0004) 0.005873	-0.0017 (-0.0025, -0.0008) 0.000156	-0.0007 (-0.0017, 0.0002) 0.144140
	60≤Aged	-0.0003 (-0.0011, 0.0006) 0.513681	-0.0005 (-0.0013, 0.0004) 0.272575	-0.0002 (-0.0011, 0.0007) 0.668748
L4 BMD	20≤Aged<30	-0.0003 (-0.0009, 0.0004) 0.413254	-0.0005 (-0.0012, 0.0001) 0.124521	0.0000 (-0.0007, 0.0007) 0.999632
	30≤Aged<40	-0.0006 (-0.0012, 0.0001) 0.084888	-0.0006 (-0.0013, -0.0000) 0.045006	-0.0001 (-0.0008, 0.0006) 0.741328
	40≤Aged<50	0.0001 (-0.0005, 0.0008) 0.679172	-0.0001 (-0.0008, 0.0005) 0.722415	0.0007 (-0.0000, 0.0014) 0.058517
	50≤Aged<60	-0.0011 (-0.0019, -0.0002) 0.016075	-0.0016 (-0.0024, -0.0007) 0.000463	-0.0004 (-0.0014, 0.0006) 0.399831
	60≤Aged	-0.0004 (-0.0012, 0.0005) 0.400768	-0.0006 (-0.0014, 0.0003) 0.176298	-0.0006 (-0.0016, 0.0003) 0.171516

Model 1: no covariates were adjusted. Model 2: race/ethnicity (Mexican American; other Hispanic; non-Hispanic white; non-Hispanic black; other races) were adjusted. Model 3: race/ethnicity (Mexican American; other Hispanic; non-Hispanic white; non-Hispanic black; other races), education level (under high school; high school or equivalent; above high school), income to poverty ratio (quartile groups), BMI (obese, overweight, normal), smoking status (less than 100 cigarettes; greater than or equal to 100 cigarettes), alcohol consumption status (had at least 12 alcohol drinks past one year; have less than 12 alcohol drinks past one year), hypertension (yes; no), diabetes (yes; no; borderline), ALT (quartile groups), AST (quartile groups), total calcium (quartile groups), cholesterol (quartile groups), and C-reactive protein (quartile groups) were adjusted. *HDL-C* high-density lipoprotein cholesterol; *BMD* bone mineral density; *ALT* alanine aminotransferase; *AST* aspartate aminotransferase

**Fig. 5:**
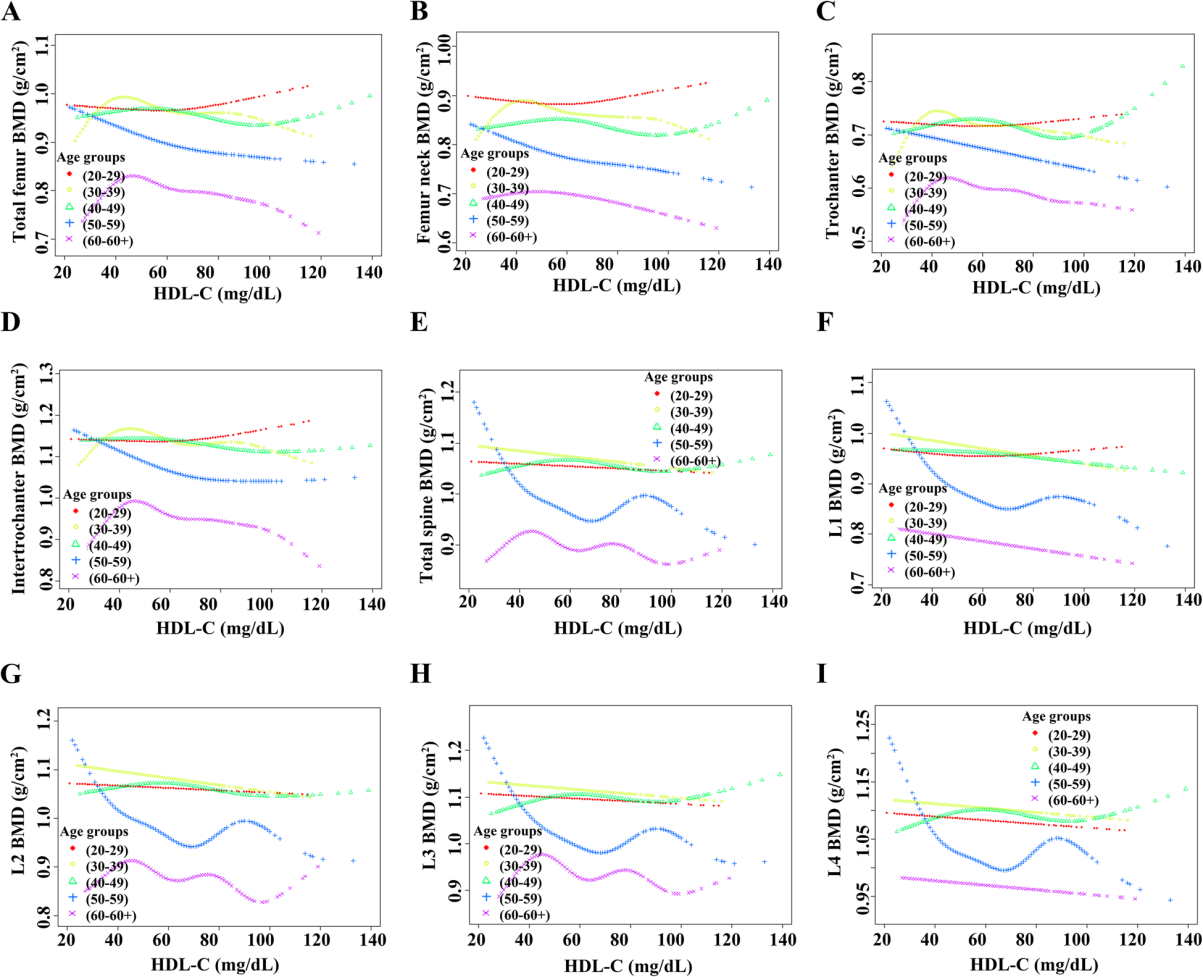
Association between HDL-C and BMD in female participants. Race/ethnicity, education level, income to poverty ratio, BMI, smoking status, alcohol consumption status, hypertension, diabetes, ALT, AST, total calcium, cholesterol, and C-reactive protein were adjusted. **a**. Total femur BMD; **b**. Femur neck BMD; **c**. Trochanter BMD; **d**. Intertrochanter BMD; **e**. Total spine BMD; **f**. L1 BMD; **g**. L2 BMD; **h**. L3 BMD; **i**. L4 BMD. Red line: 20≤Aged<30; Yellow line: 30≤Aged<40; Green line: 40≤Aged<50; Blue line: 50≤Aged<60; Purple line: 60≤Aged. HDL-C, high-density lipoprotein cholesterol; BMD, bone mineral density; ALT, alanine aminotransferase; AST, aspartate aminotransferase

For females, HDL-C levels displayed a negative association with BMD among all age groups in Model 1 and Model 2. However, when all covariates were adjusted, the results suggested that the negative association was mainly among women aged 30 to 40 or 50 to 60. Further analysis of the nonlinear relationship between HDL-C and BMD showed that (i) HDL-C levels displayed an inverted U-shaped relationship with BMD among women aged 30 to 39 or over 60 years. Moreover, the inflection points of HDL-C observed were approximately 45 mg/dL (for subjects aged 30 to 40 or subjects over 60). In addition, the two-piecewise linear regression models demonstrated that BMD rose gradually as the HDL-C level rose (HDL-C < 45 mg/dL), while no statistical significance was observed in females aged 30 to 39; BMD declined gradually as the HDL-C level rose (HDL-C > 45 mg/dL), while no statistical significance was observed in females aged over 60. (ii) HDL-C levels exhibited a U-shaped association with BMD among women aged 20 to 29 or 50 to 59 years. Moreover, the thresholds of the inflection points observed were approximately 65 mg/dL (subject aged 20 to 29) and 70 mg/dL (subject aged 50 to 59). In addition, the two-piecewise linear regression models demonstrated that BMD declined gradually with the rising HDL-C level (HDL-C less than the threshold); BMD rose gradually with the rising HDL-C level (HDL-C greater than the threshold), while no statistical significance was observed in females aged 50 to 59. The detailed results are listed in Tables 6-7 and Fig. 5.

**Table 6:** Association between HDL-C and BMD in females

		Model 1 β (95% CI) *P* value	Model 2 β (95% CI) *P* value	Model 3 β (95% CI) *P* value
Total femur BMD	20≤Aged<30	-0.0009 (-0.0014, -0.0003) 0.004084	-0.0009 (-0.0014, -0.0003) 0.003038	-0.0000 (-0.0007, 0.0006) 0.895165
	30≤Aged<40	-0.0016 (-0.0022, -0.0010) <0.000001	-0.0017 (-0.0023, -0.0011) <0.000001	-0.0010 (-0.0017, -0.0004) 0.002143
	40≤Aged<50	-0.0013 (-0.0018, -0.0008) <0.000001	-0.0013 (-0.0018, -0.0008) <0.000001	-0.0005 (-0.0010, 0.0001) 0.089961
	50≤Aged<60	-0.0020 (-0.0027, -0.0013) <0.000001	-0.0021 (-0.0028, -0.0014) <0.000001	-0.0010 (-0.0018, -0.0002) 0.013817
	60≤Aged	-0.0009 (-0.0018, -0.0001) 0.027771	-0.0011 (-0.0019, -0.0003) 0.007503	0.0000 (-0.0009, 0.0009) 0.965459
Femur neck BMD	20≤Aged<30	-0.0008 (-0.0014, -0.0002) 0.012262	-0.0008 (-0.0014, -0.0002) 0.008148	0.0002 (-0.0005, 0.0008) 0.613585
	30≤Aged<40	-0.0014 (-0.0020, -0.0008) 0.000005	-0.0015 (-0.0021, -0.0009) 0.000001	-0.0009 (-0.0016, -0.0003) 0.005558
	40≤Aged<50	-0.0012 (-0.0017, -0.0007) 0.000001	-0.0012 (-0.0017, -0.0007) <0.000001	-0.0004 (-0.0009, 0.0001) 0.127233
	50≤Aged<60	-0.0020 (-0.0027, -0.0014) <0.000001	-0.0021 (-0.0027, -0.0014) <0.000001	-0.0010 (-0.0018, -0.0003) 0.007700
	60≤Aged	-0.0009 (-0.0016, -0.0002) 0.018292	-0.0011 (-0.0018, -0.0004) 0.002431	-0.0001 (-0.0008, 0.0007) 0.863724
Trochanter BMD	20≤Aged<30	-0.0006 (-0.0011, -0.0001) 0.014098	-0.0006 (-0.0011, -0.0002) 0.010771	-0.0001 (-0.0007, 0.0004) 0.610232
	30≤Aged<40	-0.0011 (-0.0016, -0.0006) 0.000040	-0.0012 (-0.0017, -0.0006) 0.000016	-0.0007 (-0.0013, -0.0002) 0.012737
	40≤Aged<50	-0.0009 (-0.0013, -0.0004) 0.000066	-0.0009 (-0.0013, -0.0005) 0.000031	-0.0004 (-0.0008, 0.0001) 0.142166
	50≤Aged<60	-0.0014 (-0.0020, -0.0008) 0.000003	-0.0015 (-0.0021, -0.0009) 0.000001	-0.0007 (-0.0014, -0.0001) 0.030223
	60≤Aged	-0.0005 (-0.0012, 0.0002) 0.157187	-0.0007 (-0.0014, 0.0000) 0.057321	0.0002 (-0.0006, 0.0009) 0.642347
Intertrochanter BMD	20≤Aged<30	-0.0010 (-0.0017, -0.0004) 0.002288	-0.0010 (-0.0017, -0.0004) 0.001898	-0.0001 (-0.0009, 0.0006) 0.726263
	30≤Aged<40	-0.0019 (-0.0026, -0.0012) <0.000001	-0.0019 (-0.0026, -0.0012) <0.000001	-0.0012 (-0.0019, -0.0004) 0.002305
	40≤Aged<50	-0.0016 (-0.0022, -0.0010) <0.000001	-0.0016 (-0.0021, -0.0010) <0.000001	-0.0005 (-0.0012, 0.0001) 0.128057
	50≤Aged<60	-0.0024 (-0.0032, -0.0016) <0.000001	-0.0024 (-0.0033, -0.0016) <0.000001	-0.0012 (-0.0021, -0.0002) 0.016133
	60≤Aged	-0.0012 (-0.0023, -0.0002) 0.015602	-0.0014 (-0.0025, -0.0004) 0.004826	-0.0001 (-0.0011, 0.0010) 0.910292
Total spine BMD	20≤Aged<30	-0.0009 (-0.0015, -0.0004) 0.000976	-0.0010 (-0.0016, -0.0005) 0.000235	-0.0004 (-0.0010, 0.0002) 0.217578
	30≤Aged<40	-0.0011 (-0.0016, -0.0005) 0.000251	-0.0012 (-0.0017, -0.0006) 0.000042	-0.0010 (-0.0017, -0.0004) 0.001201
	40≤Aged<50	-0.0009 (-0.0014, -0.0004) 0.000360	-0.0011 (-0.0016, -0.0006) 0.000042	-0.0005 (-0.0011, 0.0001) 0.100247
	50≤Aged<60	-0.0020 (-0.0028, -0.0012) 0.000001	-0.0021 (-0.0029, -0.0013) <0.000001	-0.0012 (-0.0021, -0.0002) 0.016337
	60≤Aged	-0.0010 (-0.0019, -0.0001) 0.035685	-0.0013 (-0.0022, -0.0004) 0.004202	-0.0003 (-0.0013, 0.0006) 0.498599
L1 BMD	20≤Aged<30	-0.0009 (-0.0015, -0.0003) 0.004548	-0.0010 (-0.0015, -0.0004) 0.001433	-0.0002 (-0.0008, 0.0005) 0.640539
	30≤Aged<40	-0.0014 (-0.0020, -0.0008) 0.000012	-0.0015 (-0.0021, -0.0009) 0.000003	-0.0012 (-0.0019, -0.0005) 0.000460
	40≤Aged<50	-0.0012 (-0.0018, -0.0007) 0.000004	-0.0013 (-0.0019, -0.0008) <0.000001	-0.0007 (-0.0013, -0.0001) 0.015151
	50≤Aged<60	-0.0024 (-0.0032, -0.0016) <0.000001	-0.0025 (-0.0033, -0.0017) <0.000001	-0.0015 (-0.0025, -0.0006) 0.002117
	60≤Aged	-0.0014 (-0.0023, -0.0005) 0.002435	-0.0017 (-0.0026, -0.0008) 0.000241	-0.0006 (-0.0016, 0.0004) 0.234842
L2 BMD	20≤Aged<30	-0.0010 (-0.0015, -0.0004) 0.001734	-0.0010 (-0.0016, -0.0004) 0.000554	-0.0003 (-0.0009, 0.0004) 0.387438
	30≤Aged<40	-0.0013 (-0.0019, -0.0007) 0.000043	-0.0014 (-0.0020, -0.0008) 0.000007	-0.0012 (-0.0019, -0.0005) 0.000458
	40≤Aged<50	-0.0011 (-0.0016, -0.0005) 0.000098	-0.0012 (-0.0018, -0.0007) 0.000008	-0.0006 (-0.0012, 0.0000) 0.061290
	50≤Aged<60	-0.0020 (-0.0029, -0.0011) 0.000007	-0.0021 (-0.0030, -0.0013) 0.000002	-0.0012 (-0.0023, -0.0002) 0.022824
	60≤Aged	-0.0012 (-0.0022, -0.0003) 0.011340	-0.0016 (-0.0025, -0.0006) 0.001075	-0.0007 (-0.0017, 0.0003) 0.193459
L3 BMD	20≤Aged<30	-0.0009 (-0.0015, -0.0003) 0.001976	-0.0010 (-0.0016, -0.0005) 0.000412	-0.0004 (-0.0010, 0.0003) 0.255710
	30≤Aged<40	-0.0009 (-0.0015, -0.0003) 0.002536	-0.0011 (-0.0016, -0.0005) 0.000496	-0.0010 (-0.0017, -0.0003) 0.003036
	40≤Aged<50	-0.0007 (-0.0012, -0.0001) 0.016616	-0.0008 (-0.0013, -0.0003) 0.003383	-0.0003 (-0.0009, 0.0003) 0.335752
	50≤Aged<60	-0.0021 (-0.0030, -0.0013) 0.000002	-0.0022 (-0.0031, -0.0014) <0.000001	-0.0013 (-0.0024, -0.0003) 0.012235
	60≤Aged	-0.0010 (-0.0020, -0.0000) 0.042502	-0.0013 (-0.0023, -0.0004) 0.005913	-0.0003 (-0.0014, 0.0007) 0.548696
L4 BMD	20≤Aged<30	-0.0009 (-0.0015, -0.0004) 0.001136	-0.0010 (-0.0016, -0.0004) 0.000418	-0.0006 (-0.0013, 0.0000) 0.052108
	30≤Aged<40	-0.0008 (-0.0014, -0.0002) 0.009702	-0.0009 (-0.0015, -0.0003) 0.002916	-0.0008 (-0.0014, -0.0001) 0.018291
	40≤Aged<50	-0.0008 (-0.0014, -0.0003) 0.002988	-0.0010 (-0.0015, -0.0004) 0.000560	-0.0004 (-0.0010, 0.0002) 0.220609
	50≤Aged<60	-0.0016 (-0.0024, -0.0008) 0.000145	-0.0017 (-0.0025, -0.0009) 0.000041	-0.0008 (-0.0018, 0.0002) 0.108678
	60≤Aged	-0.0006 (-0.0016, 0.0004) 0.235832	-0.0009 (-0.0019, 0.0000) 0.053248	-0.0001 (-0.0011, 0.0010) 0.884132

Model 1: no covariates were adjusted. Model 2: race/ethnicity (Mexican American; other Hispanic; non-Hispanic white; non-Hispanic black; other races) were adjusted. Model 3: race/ethnicity (Mexican American; other Hispanic; non-Hispanic white; non-Hispanic black; other races), education level (under high school; high school or equivalent; above high school), income to poverty ratio (quartile groups), BMI (obese, overweight, normal), smoking status (less than 100 cigarettes; greater than or equal to 100 cigarettes), alcohol consumption status (had at least 12 alcohol drinks past one year; have less than 12 alcohol drinks past one year), hypertension (yes; no), diabetes (yes; no; borderline), ALT (quartile groups), AST (quartile groups), total calcium (quartile groups), cholesterol (quartile groups), and C-reactive protein (quartile groups) were adjusted. *HDL-C* high-density lipoprotein cholesterol; *BMD* bone mineral density; *ALT* alanine aminotransferase; *AST* aspartate aminotransferase

**Table 7:** Two-piecewise linear regression models of HDL-C on bone mineral density in females

Age Groups		Index			
**20≤Aged<30**		**Total femur BMD**	**Femur neck BMD**	**Trochanter BMD**	**Intertrochanter BMD**
	Fitting by the standard linear model	-0.0000 (-0.0007, 0.0006) 0.8952	0.0002 (-0.0005, 0.0008) 0.6136	-0.0001 (-0.0007, 0.0004) 0.6102	-0.0001 (-0.0009, 0.0006) 0.7263
	Fitting by the two-piecewise linear model				
	Inflection point (mg/dL)	65	65	65	65
	HDL-C < Infection point	-0.0011 (-0.0021, -0.0001) 0.0257	-0.0006 (-0.0016, 0.0004) 0.2539	-0.0011 (-0.0019, -0.0002) 0.0141	-0.0013 (-0.0024, -0.0002) 0.0228
	HDL-C > Infection point	0.0016 (0.0003, 0.0028) 0.0170	0.0013 (-0.0000, 0.0026) 0.0549	0.0013 (0.0001, 0.0024) 0.0291	0.0016 (0.0002, 0.0031) 0.0298
	Log likelihood ratio	0.004	0.05	0.004	0.006
**30≤Aged<40**		**Total femur BMD**	**Femur neck BMD**	**Trochanter BMD**	**Intertrochanter BMD**
	Fitting by the standard linear model	-0.0010 (-0.0017, -0.0004) 0.0021	-0.0009 (-0.0016, -0.0003) 0.0056	-0.0007 (-0.0013, -0.0002) 0.0127	-0.0012 (-0.0019, -0.0004) 0.0023
	Fitting by the two-piecewise linear model				
	Inflection point (mg/dL)	45	45	45	45
	HDL-C < Infection point	0.0021 (-0.0007, 0.0048) 0.1389	0.0013 (-0.0014, 0.0041) 0.3443	0.0018 (-0.0006, 0.0043) 0.1444	0.0030 (-0.0002, 0.0062) 0.0626
	HDL-C > Infection point	-0.0015 (-0.0023, -0.0007) 0.0001	-0.0013 (-0.0020, -0.0005) 0.0013	-0.0012 (-0.0018, -0.0005) 0.0012	-0.0018 (-0.0027, -0.0009) <0.0001
	Log likelihood ratio	0.02	0.092	0.031	0.007
**50≤Aged<60**		**Total femur BMD**	**Femur neck BMD**	**Trochanter BMD**	**Intertrochanter BMD**
	Fitting by the standard linear model	-0.0010 (-0.0018, -0.0002) 0.0138	-0.0010 (-0.0018, -0.0003) 0.0077	-0.0007 (-0.0014, -0.0001) 0.0302	-0.0012 (-0.0021, -0.0002) 0.0161
	Fitting by the two-piecewise linear model				
	Inflection point (mg/dL)	70	70	70	70
	HDL-C < Infection point	-0.0025 (-0.0037, -0.0012) 0.0002	-0.0017 (-0.0030, -0.0005) 0.0073	-0.0015 (-0.0026, -0.0004) 0.0085	-0.0032 (-0.0047, -0.0017) <0.0001
	HDL-C > Infection point	0.0008 (-0.0007, 0.0022) 0.2960	-0.0002 (-0.0016, 0.0012) 0.7467	0.0001 (-0.0011, 0.0014) 0.8311	0.0013 (-0.0004, 0.0030) 0.1407
	Log likelihood ratio	0.003	0.161	0.084	<0.001
		**L1 BMD**	**L2 BMD**	**L3 BMD**	**L4 BMD**
	Fitting by the standard linear model	-0.0015 (-0.0025, -0.0006) 0.0021	-0.0012 (-0.0023, -0.0002) 0.0228	-0.0013 (-0.0024, -0.0003) 0.0122	-0.0008 (-0.0018, 0.0002) 0.1087
	Fitting by the two-piecewise linear model				
	Inflection point (mg/dL)	70	70	70	70
	HDL-C < Infection point	-0.0029 (-0.0044, -0.0013) 0.0004	-0.0030 (-0.0047, -0.0013) 0.0006	-0.0035 (-0.0052, -0.0018) <0.0001	-0.0025 (-0.0041, -0.0009) 0.0023
	HDL-C > Infection point	0.0001 (-0.0016, 0.0019) 0.8969	0.0009 (-0.0010, 0.0028) 0.3502	0.0013 (-0.0006, 0.0031) 0.1889	0.0012 (-0.0006, 0.0030) 0.1869
	Log likelihood ratio	0.025	0.007	<0.001	0.007
**60≤Aged**		**Total femur BMD**	**Femur neck BMD**	**Trochanter BMD**	**Intertrochanter BMD**
	Fitting by the standard linear model	0.0000 (-0.0009, 0.0009) 0.9655	-0.0001 (-0.0008, 0.0007) 0.8637	0.0002 (-0.0006, 0.0009) 0.6423	-0.0001 (-0.0011, 0.0010) 0.9103
	Fitting by the two-piecewise linear model				
	Inflection point (mg/dL)	45	45	45	45
	HDL-C < Infection point	0.0076 (0.0031, 0.0121) 0.0010	0.0056 (0.0017, 0.0095) 0.0050	0.0055 (0.0017, 0.0094) 0.0052	0.0096 (0.0041, 0.0151) 0.0007
	HDL-C > Infection point	-0.0008 (-0.0018, 0.0002) 0.1095	-0.0007 (-0.0015, 0.0002) 0.1171	-0.0004 (-0.0013, 0.0004) 0.3462	-0.0011 (-0.0023, 0.0001) 0.0716
	Log likelihood ratio	<0.001	0.003	0.004	<0.001

Race/ethnicity (Mexican American; other Hispanic; non-Hispanic white; non-Hispanic black; other races), education level (under high school; high school or equivalent; above high school), income to poverty ratio (quartile groups), BMI (obese, overweight, normal), smoking status (less than 100 cigarettes; greater than or equal to 100 cigarettes), alcohol consumption status (had at least 12 alcohol drinks past one year; have less than 12 alcohol drinks past one year), hypertension (yes; no), diabetes (yes; no; borderline), ALT (quartile groups), AST (quartile groups), total calcium (quartile groups), cholesterol (quartile groups), and C-reactive protein (quartile groups) were adjusted. *HDL-C* high-density lipoprotein cholesterol; *BMD* bone mineral density; *ALT* alanine aminotransferase; *AST* aspartate aminotransferase

### Relationship between HDL-C levels and bone loss

To explore whether HDL-C had potential value in predicting osteopenia or osteoporosis, the female participants were subdivided into three groups (low HDL-C tertile: 21-48 mg/dL; middle HDL-C tertile: 49-61 mg/dL; high HDL-C tertile: 62-139 mg/dL). Since the sample size of the osteoporosis or osteopenia participants was much smaller than that of the normal BMD group after weighing, the OR value and 95% CI could not be calculated; thus, the sample numbers were not weighted in this analysis. After adjusting for confounders, compared with the participants with middle HDL-C levels (49-61 mg/dL), females with high HDL-C levels (62-139 mg/dL) had an increased risk of osteopenia or osteoporosis, especially women aged 40-59. In addition, females aged 40-49 who had low HDL-C levels (21-48 mg/dL) also had a high incidence of osteopenia or osteoporosis. The specific results are listed in **Table** **8**.

**Table 8:** Associations between HDL-C and osteopenia or osteoporosis in females

	Age (20-29)	Age (30-39)	Age (40-49)	Age (50-59)	Age (60-)	Total
Non-adjusted						
HDL (49-61 mg/dL)	Reference	Reference	Reference	Reference	Reference	Reference
HDL (21-48 mg/dL)	0.9387 (0.5986, 1.4718) 0.782692	0.5797 (0.3550, 0.9466) 0.029321	1.9966 (1.2513, 3.1860) 0.003729	0.9575 (0.5839, 1.5700) 0.863251	0.8372 (0.5043, 1.3900) 0.492132	0.9872 (0.7972, 1.2224) 0.905687
HDL (62-139 mg/dL)	1.0672 (0.6872, 1.6575) 0.772075	1.3464 (0.8806, 2.0587) 0.169779	2.5978 (1.6734, 4.0328) 0.000021	2.7259 (1.6882, 4.4014) 0.000041	1.5604 (0.9488, 2.5663) 0.079599	1.7096 (1.4004, 2.0869) <0.000001
Adjusted						
HDL (49-61 mg/dL)	Reference	Reference	Reference	Reference	Reference	Reference
HDL (21-48 mg/dL)	1.2054 (0.7176, 2.0251) 0.480194	0.6875 (0.3996, 1.1829) 0.175989	2.4873 (1.4866, 4.1618) 0.000521	1.0997 (0.6186, 1.9549) 0.746046	1.1149 (0.5826, 2.1335) 0.742546	1.1529 (0.9135, 1.4550) 0.230883
HDL (62-139 mg/dL)	0.9365 (0.5670, 1.5466) 0.797616	1.1742 (0.7322, 1.8830) 0.505203	2.0781 (1.2673, 3.4075) 0.003744	2.3301 (1.3215, 4.1085) 0.003463	1.5008 (0.7983, 2.8214) 0.207419	1.3831 (1.1118, 1.7207) 0.003602

Race/ethnicity (Mexican American; other Hispanic; non-Hispanic white; non-Hispanic black; other races), education level (under high school; high school or equivalent; above high school), income to poverty ratio (quartile groups), BMI (obese, overweight, normal), smoking status (less than 100 cigarettes; greater than or equal to 100 cigarettes), alcohol consumption status (had at least 12 alcohol drinks past one year; have less than 12 alcohol drinks past one year), hypertension (yes; no), diabetes (yes; no; borderline), ALT (quartile groups), AST (quartile groups), total calcium (quartile groups), cholesterol (quartile groups), and C-reactive protein (quartile groups) were adjusted. *HDL-C*, high-density lipoprotein cholesterol; *ALT* alanine aminotransferase; *AST* aspartate aminotransferase

## Discussion

In the present study, HDL-C displayed a negative correlation with BMD, especially in females. Moreover, a nonlinear relationship between HDL-C and BMD was observed among females across different age ranges. Additionally, females with high HDL-C levels had an increased incidence of osteopenia or osteoporosis, which suggests that HDL-C levels might have potential predictive value for osteopenia or osteoporosis.

Previous studies have explored the relationship between HDL-C and BMD [17–20]. For example, in Iranian women, Maghbooli et al. found that HDL-C levels displayed an inversely correlation with BMD in postmenopausal women with vitamin D deficiency [17]. Zhang et al. demonstrated that HDL-C exhibited a negative association with BMD in Chinese women above 50 years of age [18]. Makovey et al. observed a modest inverse relationship between hip BMD and HDL-C in postmenopausal women [31]. Jeong et al. found that HDL-C levels displayed a positive correlation with BMD in postmenopausal women, but the positive correlation was too weak (β < 0.001) [20]. Cui et al. demonstrated that HDL-C levels were not linked to BMD in pre- or postmenopausal women [19]. In summary, the conclusions remain controversial, and these studies had limitations, such as a small sample size, a selected population, or adjusted variables; however, the present study avoids these shortcomings. First, this study used a nationally representative sample from the NHANES database, which allowed a huge sample size. Second, since previous studies usually considered the relationship between HDL-C and BMD in females, especially postmenopausal females, the present study also considered the potential impact of gender and age. Third, this study adjusted for more variables that might potentially influence BMD. As expected, here, this study demonstrated not only a correlation between HDL-C and BMD but also a potential predictive value of HDL-C for osteoporosis or osteopenia.

The mechanisms underlying the correlation between HDL-C and BMD are uncertain. Especially in basic research, there is no robust evidence that supports this negative association. According to related studies, several possible factors might account for this phenomenon. First, HDL-C, especially at a high level, affects BMD through sex hormones. There are already a large number of studies demonstrating that sex hormones, including androgen and oestrogen, play essential roles in maintaining bone balance [32–34]. Semmens et al. found that testosterone levels present a strong negative association with HDL-C levels [35]. Jirapinyo et al. observed that combined oral oestrogen/progestogen increased BMD in postmenopausal women but decreased HDL-C levels [36]. In the present study, a difference in the association between HDL-C and BMD was observed among different gender and age groups, which suggests that hormone levels, especially sex hormones, contribute to the association. However, because the NHANES database 2005-2010 did not collect information on the levels of sex hormones, the sex hormone levels could not be described in the present study. Second, high HDL-C levels might affect BMD by activating an inflammatory reaction. There is already evidence suggesting that inflammatory factors can affect bone metabolism, such as influencing the activation or function of osteoclasts [37, 38], which might be a possible pathway by which high HDL-C levels affect BMD. For example, Mazidi et al. found that HDL-C was positively associated with inflammatory indicators, such as C-reactive protein, white blood cells, and fibrinogen, in adults [39]. However, there is no direct evidence supporting this hypothesis; thus, further experiments are necessary.

The present study not only demonstrates a negative association between HDL-C and BMD but also has certain clinical value that can guide clinicians. Specifically, the negative association suggests that subjects with a higher HDL-C level might have a lower BMD. This study found that females with high HDL-C levels had an increased incidence of osteopenia or osteoporosis. However, it is important to note that although the associations were different according to ages, there may be no clinical implications in some age groups. The results of multiple logistic regression models shows that the females aged 20 to 39 or over 60 with high HDL-C levels did not have a high prevalence of osteoporosis or osteopenia (P > 0.05). Therefore, these findings suggest that clinicians should be alert to the risk of reduced bone mass for individuals with high HDL-C levels, especially postmenopausal women. For these patients, close monitoring of BMD and early intervention may be necessary. In addition, osteoporotic fracture is one of the most common and serious complications for patients with osteoporosis [8]. Therefore, future research is warranted to explore whether high HDL-C levels can indicate an increased risk of osteoporotic fracture.

For a long time, numerous researchers and studies have believed that HDL-C is beneficial to health [40, 41]. Especially in the field of cardiovascular disease [2, 4], HDL-C is considered to be negatively correlated with adverse cardiovascular events [2–5]. However, numerous research results have indicated that the contribution of HDL-C to human health might be highly overestimated. Several years ago, it was demonstrated that drugs that increased HDL-C did not prevent adverse cardiovascular events [42]. Other recent studies have reported that HDL-C displays an inverted U-shaped relationship with all-cause mortality [7, 43]. All of these findings indicate that elevated HDL-C levels may be detrimental to health and may even cause certain adverse events. This study established that HDL-C exhibits an inverse relationship with BMD in adult females, corroborating this view. In addition, it is worth mentioning that most basic studies usually focus on the impact of low HDL-C but not high HDL-C on bone metabolism [44, 45]. Although many studies have demonstrated that low HDL-C levels can affect bone metabolism through a variety of pathways, there is no evidence to elucidate the impact of high HDL-C levels on bone metabolism, especially the function and activity of osteoblasts and osteoclasts. As a result, future research should focus on the specific mechanism underlying the effect of elevated HDL-C levels on bone metabolism, which is necessary for improving theoretical knowledge of the impact of lipid metabolism on bone balance.

### Strength and study limitation

This study has several strengths for studying the association between HDL-C and BMD. (i) This study was based on data in the NHANES database, which has a large sample size and adequate clinical information. (ii) This study estimated the difference in the association between HDL-C and BMD in diverse populations by stratifying age and sex. (iii) In addition to the linear relationship between HDL-C and BMD, this study also employed statistical analyses assessing a nonlinear model. (iv) This study found that female participants with higher HDL-C levels had an increased incidence of osteopenia or osteoporosis, which suggests that HDL-C might have potential value for predicting osteopenia or osteoporosis. In addition, this study has some limitations: (i) This study is based on American participants. Because of the differences in genetic, lingual, cultural, and environmental factors, it is uncertain whether the association between HDL-C and BMD applies to other countries or races. (ii) Because some related information, such as sex hormone and parathyroid hormone levels, was not provided in the NHANES database 2005-2010, this study could not describe these conditions in current cases. (iii) The questionnaire data were collected through questionnaires and interviews, which may lead to recall bias and potentially affect the research conclusion. (iv) Because of the cross-sectional study design, the causal involvement of HDL-C and BMD could not be confirmed. Moreover, there may be some potential confounding factors that were not adjusted.

## Conclusion

This study demonstrated that HDL-C levels exhibit an inverse correlation with BMD. Especially in females, clinicians need to be alert to patients with high HDL-C levels, which may indicate an increased risk of osteoporosis or osteopenia. For these patients, close monitoring of BMD and early intervention may be necessary.

## Supplementary Information

**Additional file 1: Table S1.** Definition of osteoporosis and osteopenia.

## Data Availability

The datasets obtained and analysed during the current study are available in the NHANES [https://www.cdc.gov/nchs/nhanes/index.htm].
