# Supplementary material for: High-density Lipoprotein Cholesterol Is Negatively Correlated with Bone Mineral Density and Has Potential Predictive Value for Bone Loss
Source: Lipids Health Dis. 2021 Jul 25;20:75. doi: 10.1186/s12944-021-01497-7 (PMC8310606; doi:10.1186/s12944-021-01497-7)
Supplement: Supplementary file 1 — Additional file 1: Table S1. Definition of osteoporosis and osteopenia. [file 12944_2021_1497_MOESM1_ESM.docx]

**Table S1** Definition of osteoporosis and osteopenia.

|  | Osteopenia | Osteoporosis |
| --- | --- | --- |
| Men |  |  |
| Total Femur (g/cm^2^) | 0.68–0.90 | < 0.68 |
| Femur Neck (g/cm^2^) | 0.59–0.79 | < 0.59 |
| Trochanter (g/cm^2^) | 0.49–0.66 | < 0.49 |
| Intertrochanter (g/cm^2^) | 0.78–1.03 | < 0.78 |
| Female |  |  |
| Total Femur (g/cm^2^) | 0.64–0.82 | < 0.64 |
| Femur Neck (g/cm^2^) | 0.56–0.74 | < 0.56 |
| Trochanter (g/cm^2^) | 0.46–0.61 | < 0.46 |
| Intertrochanter (g/cm^2^) | 0.74–0.95 | < 0.74 |
